# Impact of stack pressure on coulometric titration time analysis

**DOI:** 10.1038/s42004-025-01496-0

**Published:** 2025-04-01

**Authors:** Jaka Sivavec, Kostiantyn V. Kravchyk, Maksym V. Kovalenko

**Affiliations:** 1 Laboratory for Thin Films and Photovoltaics, Empa – Swiss Federal Laboratories for Materials Science and Technology, Überlandstrasse 129, CH-8600 Dübendorf, Switzerland; 2 Laboratory of Inorganic Chemistry, Department of Chemistry and Applied Biosciences, ETH Zürich, Vladimir-Prelog-Weg 1, CH-8093 Zürich, Switzerland; 3 SKKU Institute of Energy Science and Technology (SIEST), Sungkyunkwan University (SKKU), 2066, Seobu-ro, Jangan-gu, Suwon, Gyeonggi-do 16419 Republic of Korea

**Keywords:** Batteries, Batteries, Analytical chemistry

## Abstract

Coulometric titration time analysis (CTTA) has recently been introduced as a quantitative electrochemical technique to assess the compatibility of Li-ion electrolytes with metallic lithium. Here, the authors outline that the applied stack pressure is a critical factor in influencing CTTA results, highlighting the need for careful stack pressure assessment to perform conclusive CTTA experiments.

Studying the Li/solid-state electrolyte (SSE) interface is crucial for developing and eventually commercializing Li metal or anode free solid-state batteries^1^. To investigate the processes occurring at the SSE surface upon contact with metallic Li, researchers have employed a variety of surface-sensitive techniques, such as X-ray photoelectron spectroscopy (XPS)^2,3^, time-of-flight secondary ion mass spectrometry (ToF-SIMS)^4^, scanning electron microscopy (SEM)^5^, transmission electron microscopy (TEM)^6,7^, and X-ray micro-computed tomography (micro-CT)^8,9^ among many others. Although these advanced techniques are crucial for uncovering the chemical processes at the SSE/Li interface, they often require dedicated experimental setups, which limits their applicability to a smaller number of studied samples. From this perspective, a readily accessible, fast, and accurate assessment of the compatibility between any SSE and Li metal is imperative.

In the context of accurate quantification of the Li/SSE interfacial reactions, a new technique called coulometric titration time analysis (CTTA) has recently been introduced^10^. CTTA operates via a repeated sequence of Li deposition steps, each separated by a waiting period during which the freshly plated Li metal reacts with the SSE. A schematic of this process is shown in Fig. 1a, with a typical voltage profile displayed in Fig. 1b. In short, CTTA measurements are performed using a Li/SSE/CC half-cell, where an SSE pellet is sandwiched between a Li-based counter/reference electrode (e.g., Li metal or Li-In alloy) and a current collector (CC) foil acting as the working electrode. During the initial charging step, Li metal is plated onto the CC, initiating the reaction with the SSE. This process forms a symmetrical cell (Li/SSE/Li/CC), causing the cell potential to approach 0 V vs. Li^+^/Li and remain stable until the plated Li is fully consumed. This completion point, marked by a sharp rise in the cell potential, signals the start of the next Li deposition step. This approach ensures a constant supply of Li at the CC and quantifies the amount of Li consumed in the reaction by measuring the total charge transferred during the experiment (Fig. 1c). As demonstrated in the pioneering work by Janek et al.^10^, CTTA can be applied to various SSEs and provides key information about the stability of the formed Li/SSE interface. Additionally, by combining CTTA data with XPS analysis^11^, which identifies the chemical products of the interfacial reaction, it is possible to assess the thickness of the solid electrolyte interface (SEI) layer. For example, in the case of Li_6_PS_5_Cl (LPSCl) SSE, the authors estimated an SEI thickness of *ca*. 315 nm after one week of contact with Li metal^10^, assuming the formation of a planar, non-porous SEI layer, which is in agreement with estimates based on recent ToF-SIMS measurements^4^.

**Fig. 1:**
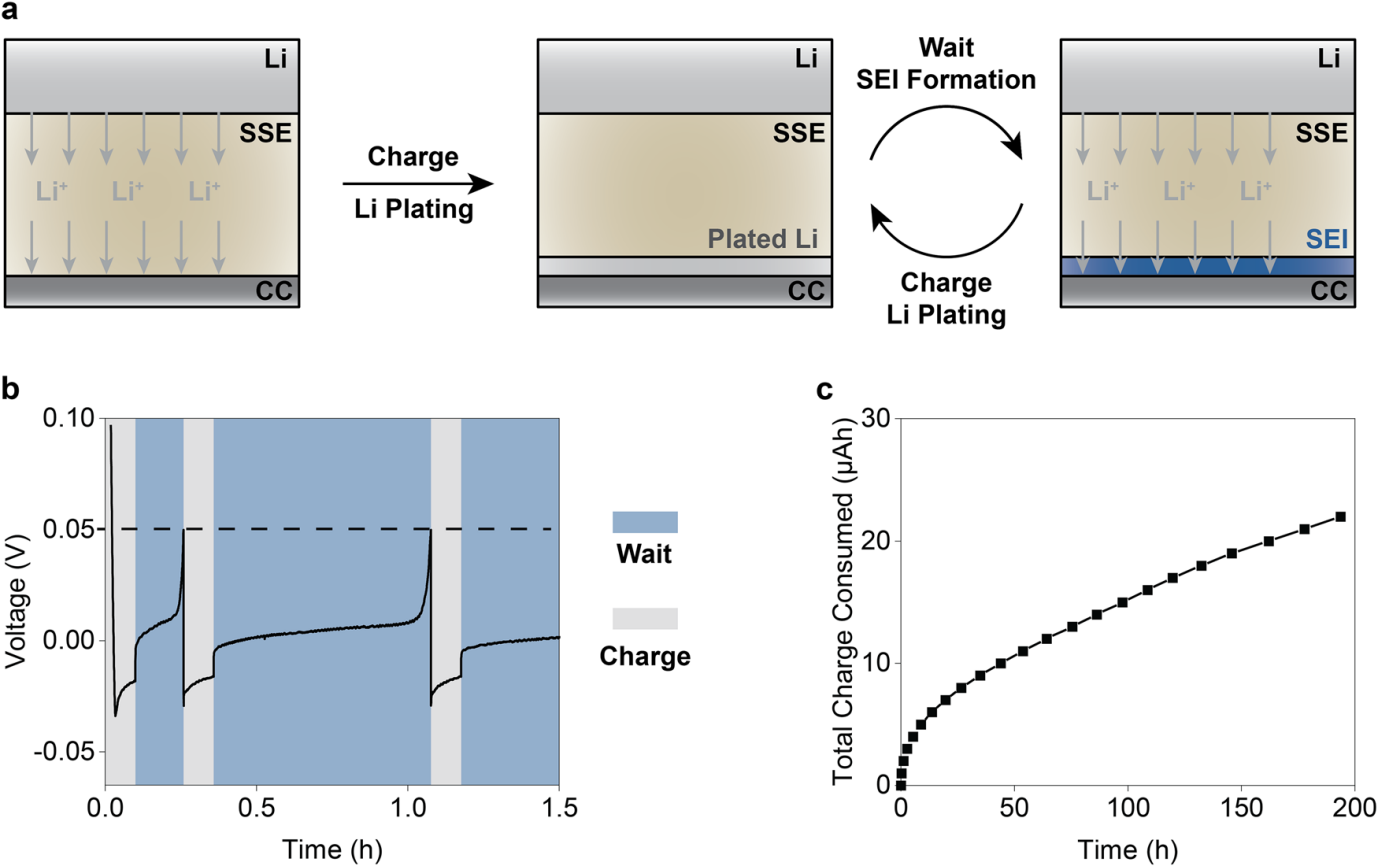
Outline of the CTTA method. **a** A schematic representation of a coulometric titration time analysis (CTTA) experiment. The electrochemical cell used in the experiment consists of a Li metal anode, a solid-state electrolyte (SSE) separator, and a metallic current collector (CC) The formation of a solid-electrolyte interface (SEI) is shown in blue. **b** A typical voltage profile recorded during the measurement. **c** Cumulative charge consumed in the reaction between the SSE and metallic Li over time.

Although this method may appear straightforward, the data obtained can vary significantly depending on the applied conditions, potentially leading to incorrect conclusions regarding the reactivity of a given SSE with metallic Li. Incorrect conclusions could lead to misguided research directions, wasted resources, and potentially unsafe battery designs. Key factors that can influence the measurement include applied current density, capacity limitation of the plating step, temperature, and the current collector material, all of which the authors of the pioneering work describe in detail. In this manuscript, we aim to highlight another critical factor, cycling stack pressure, which has a substantial impact on the obtained results.

To demonstrate the dependence of the measured charge consumption on stack pressure, we prepared Li/SSE/stainless steel “anode-free” cells based on LPSCl SSE and conducted CTTA measurements at different stack pressures of 1.7 MPa, 13 MPa, and 30 MPa. All measurements were performed using a 10-µm thick stainless steel foil as the current collector at room temperature. A current of 10 µA was applied with a capacity limitation of 1 µAh for each Li transfer step. Our results indicate that the rate of Li consumption at the interface is strongly influenced by changes in stack pressure (Fig. 2a), which can be explained by variations in the effective contact area under different pressures. At 1.7 MPa, Li plating occurs only on a fraction of the CC surface; coverage improves significantly at 13 MPa and is nearly complete at 30 MPa. This trend is also evident from the optical images of the current collector surface taken after the measurements (Fig. 2b–d) as compared to the pristine current collector surface (Supplementary Fig. S1).

**Fig. 2:**
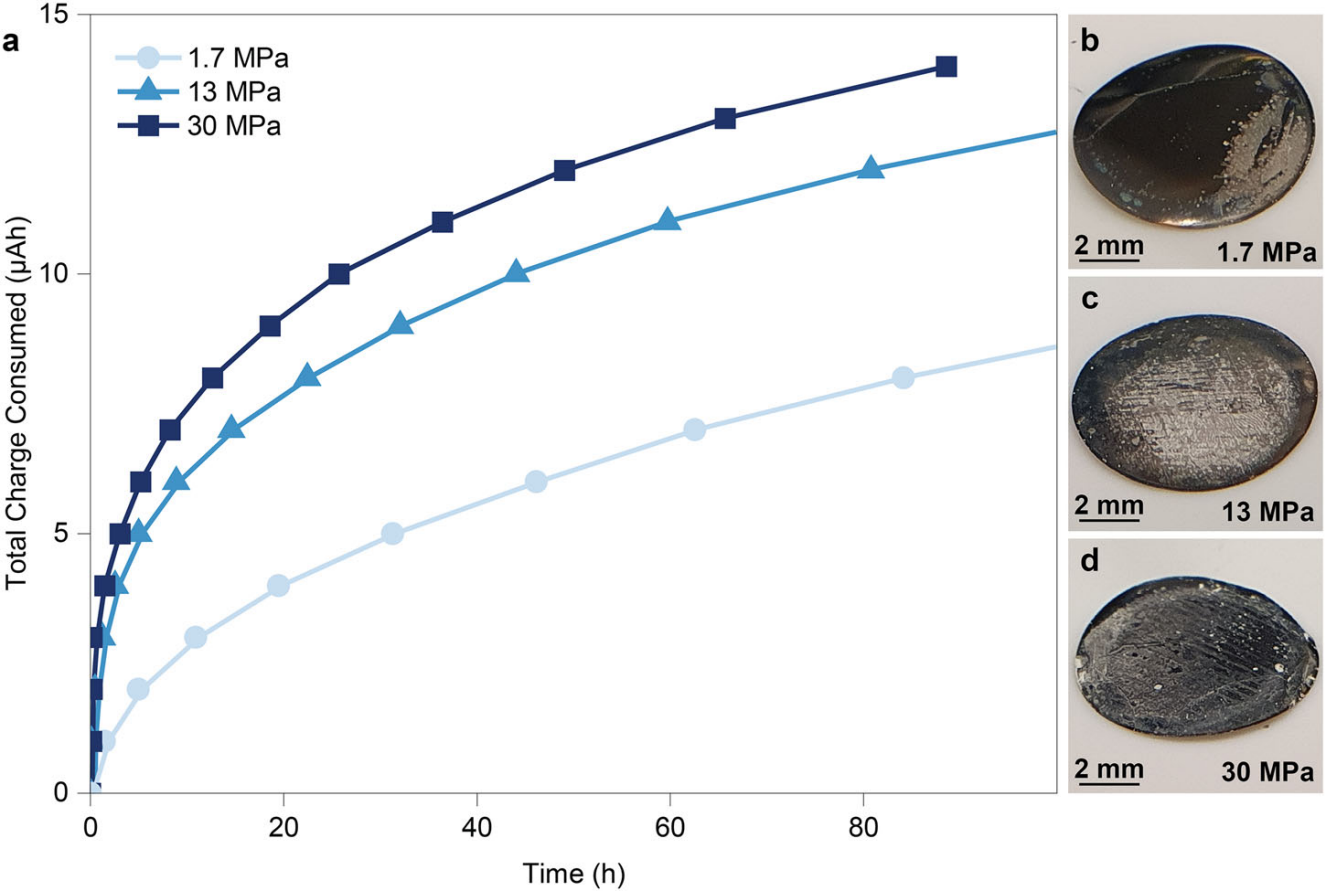
Effects of stack pressure variation. **a** The charge consumed in the side reaction between LPSCl and metallic Li versus time at different stack pressures. Optical photographs of the current collectors after 100 h of measurement at stack pressures of 1.7 MPa (**b**), 13 MPa (**c**), and 30 MPa (**d**).

The extent to which stack pressure will affect the measurement results can vary based on different cell holder setups, mechanical properties of the current collector, its thickness, and the mechanical properties of the SSE. It is expected that the exact stack pressure at which sufficient contact area is achieved could vary significantly between different experimental setups. Therefore, we recommend conducting preliminary studies on stack pressure effects for each specific system. A pressure that achieves satisfactory Li coverage should be identified and then consistently applied for all subsequent measurements to ensure the reliability of the CTTA protocol. Notably, the accessible surface area for Li plating can also be increased if the SSE is densified together with the CC in a dedicated pressing die setup, which can also act as a cell holder for electrochemical measurements and was also used in the original study^10^. Using this approach, intimate contact between the SSE and the current collector is established during the densification step and is maintained throughout the CTTA measurement, minimizing the effects of sample handling and ensuring greater consistency and reliability of the results. Further improving the cell holder, for example, by carefully polishing the stainless steel CC, can also ensure that satisfactory Li coverage is obtained at reasonably low stack pressures, in order to avoid Li penetration into the SSE separator.

In summary, this comment underscores the necessity of carefully assessing the effects of stack pressure before performing CTTA experiments. As shown for LPSCl SSE, cycling stack pressure can have a significant effect on the accessible surface area for Li plating, greatly influencing the observed reaction rate between Li and the SSE. Therefore, an optimal pressure should be identified, and consistently applied, to ensure reliability and comparability of the results. A similar dependency is expected for other solid-electrolyte systems, though the degree of this effect will vary based on the mechanical properties of a given SSE.

## Supplementary information

Supplementary Information
