# Supplementary material for: Impact of stack pressure on coulometric titration time analysis
Source: Commun Chem. 2025 Apr 1;8:96. doi: 10.1038/s42004-025-01496-0 (PMC11962158; doi:10.1038/s42004-025-01496-0)
Supplement: Supplementary file 1 — Supplementary Information [file 42004_2025_1496_MOESM1_ESM.pdf]

Supporting Information for

Impact of Stack Pressure on Coulometric Titration Time Analysis

Jaka Sivavec,[1,2] Kostiantyn V. Kravchyk,[1,2] Maksym V. Kovalenko[1,2,3*]*

[1] Laboratory for Thin Films and Photovoltaics, Empa – Swiss Federal Laboratories for Materials Science and Technology, Überlandstrasse 129, CH-8600 Dübendorf, Switzerland

[2] Laboratory of Inorganic Chemistry, Department of Chemistry and Applied Biosciences, ETH Zürich, Vladimir-Prelog-Weg 1, CH-8093 Zürich, Switzerland

[3]SKKU Institute of Energy Science and Technology (SIEST), Sungkyunkwan University (SKKU), 2066, Seobu-ro, Jangan-gu, Suwon, Gyeonggi-do 16419, Republic of Korea

Corresponding Authors:

*E-mails: mvkovalenko@ethz.ch and Kostiantyn.Kravchyk@empa.ch

Preparation and Assembly of Electrochemical Cells

The Li/LPSCl/stainless steel cells were prepared by first pressing 100 mg of LPSCl (KRI) inside an 8 mm pressing die at 2 t (390 MPa) for 3 min. The LPSCl pellet was then removed from the pressing die and transferred to a dedicated cell holder (Sphere). An 8 mm Li disc and an 8 mm stainless steel current collector were placed on opposite sides of the LPSCl pellet and a pressure of 75 MPa was applied for 3 min. After this the pressure was reduced to the specified cycling pressure.

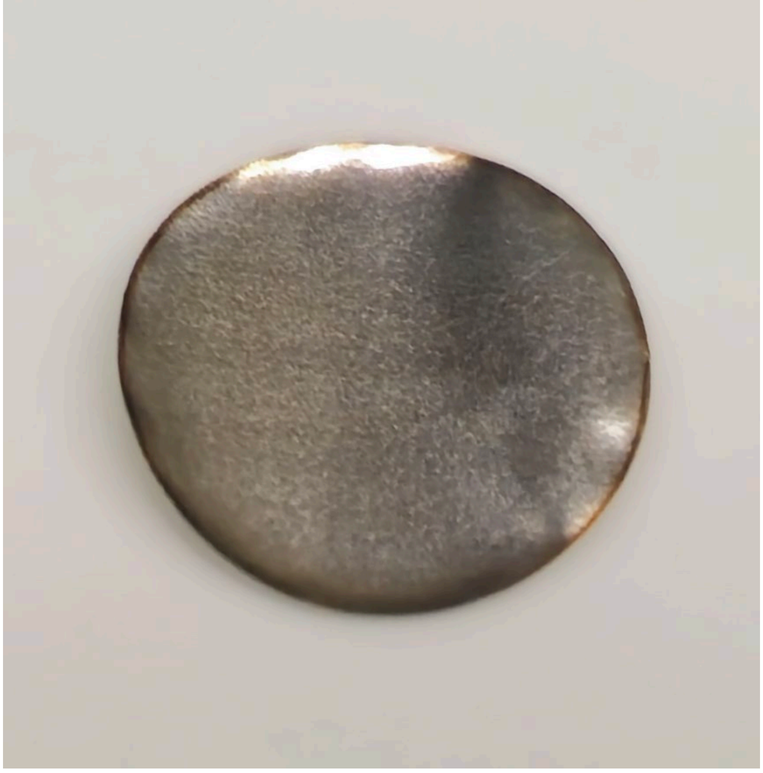

Figure S1. Optical photograph of a pristine stainless steel current collector. The diameter of the disc is equal to 8 mm.
